# Changes in Morbidity, Physical Fitness, and Perceived Quality of Life among Schoolchildren following Four Years of Different Mass Drug Administration Strategies against *Schistosoma mansoni* Infection in Mwanza Region, Northwestern Tanzania

**DOI:** 10.4269/ajtmh.19-0428

**Published:** 2019-11-11

**Authors:** Annette Olsen, Safari Kinung’hi, Godfrey Kaatano, Pascal Magnussen

**Affiliations:** 1 Section for Parasitology and Aquatic Pathobiology, Faculty of Health and Medical Sciences, University of Copenhagen, Copenhagen, Denmark;; 2 National Institute for Medical Research, Mwanza Research Centre, Mwanza, Tanzania;; 3 Centre for Medical Parasitology, Faculty of Health and Medical Sciences, University of Copenhagen, Copenhagen, Denmark

## Abstract

*Schistosoma mansoni* infection negatively impacts children’s physical health and may influence general well-being. Schistosomiasis control programs aim at reducing morbidity through mass drug administration (MDA). This study aimed to compare morbidity markers between two cohorts of Tanzanian schoolchildren with initial high prevalence of *S. mansoni* infection. One cohort (*N* = 254 at baseline) received annual MDA for 4 years using community-wide treatment (CWT). The second cohort (*N* = 318 at baseline) received school-based treatment (SBT) every other year for 4 years. At year 5, the CWT cohort and the SBT cohort were reduced to 153 and 221 children, respectively. The characteristics of the 198 children lost to follow-up did not differ at baseline from those who were examined in year 5. *Schistosoma mansoni* infection*,* hemoglobin (Hb) and anemia, physical fitness, and perceived quality of life were investigated at baseline, year 3, and year 5, whereas liver and spleen pathology (ultrasound) were investigated only at baseline and year 5. Cohorts were compared using two-way mixed-model analysis of variance (ANOVA). Both treatment regimens significantly decreased individual-level mean intensity of *S. mansoni* infection, anemia, and hepatomegaly, and increased Hb levels after 5 years. Hepatomegaly was the only parameter affected by the treatment regimen as the CWT approach reduced the percentage of individuals with hepatomegaly significantly more than the SBT approach. Both treatment regimens led to reduced physical fitness at year 5 compared with baseline. The modest impact of the two control strategies are probably due to initial low intensity of infection, ensuring low level of schistosomiasis-related morbidity.

## INTRODUCTION

Schistosomiasis is one of the neglected tropical diseases and a major public health problem, especially in sub-Saharan Africa.^1,2^ Of the 252 million estimated cases of schistosomiasis, more than 90% occur in sub-Saharan Africa.^3^ Ten years ago, the United Republic of Tanzania had the second highest number of cases (19 million) in the region.^2^

In Tanzania, both intestinal and urogenital schistosomiases are endemic, but along the shores of Lake Victoria, the intestinal form caused by *Schistosoma mansoni* is the most common. Although the infection occurs in adolescents and adults in these communities, it is especially prevalent in school-aged children.^4,5^

The clinical consequences of *S. mansoni* infections result from tissue damage and blood loss caused by eggs trapped in host tissues. The immunologic reaction to the eggs causes granuloma formation in the intestine and liver, leading to liver enlargement in advanced cases. This is frequently associated with portal hypertension and may result in enlargement of the spleen. Chronic infections, particularly in children, lead to marked nonspecific symptoms such as anemia, malnutrition, impaired growth, impaired mental development, and general body weakness.^1^

Presently, control of schistosomiasis focuses on treatment with praziquantel of the target population, through mass drug administration (MDA) programs.^6^ The Schistosomiasis Consortium for Operational Research and Evaluation (SCORE) project (https://score.uga.edu/) aimed to develop optimal approaches to control schistosomiasis by comparing the different MDA schedules. In a number of randomized intervention trials, different levels of treatment efforts have measured changes in prevalence and intensity of schistosomiasis in schoolchildren, and the result of this effort in Mwanza Region, Tanzania, is presented elsewhere.^7^

**Figure 1.**
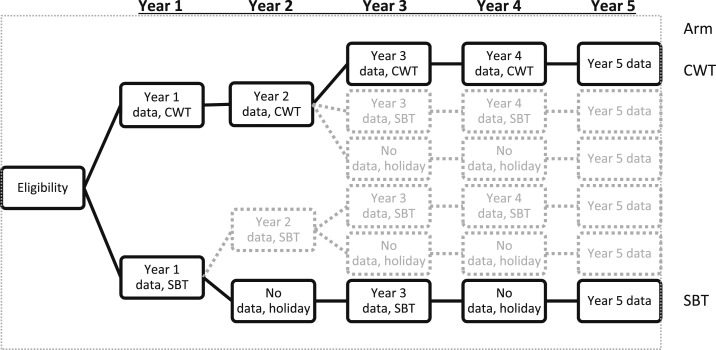
The two-armed cohort study (arms in bold) was nested in a larger cross-sectional study. The cohort study investigated the effects of the most intense level of treatment (CWT) and the less-intense treatment strategy (SBT) on subtle morbidity. CWT = community-wide treatment; SBT = school-based treatment; holiday indicates years in which a village did not receive mass drug administration with praziquantel.

The current aim of the schistosomiasis control is to reduce morbidity at the individual level. Thus, the SCORE project also investigated changes in *S. mansoni*–associated morbidity in a nested cohort study within the intervention trials (Figure 1). The two intervention arms with the highest and lowest levels of treatment efforts, respectively, were selected, and schoolchildren in these arms were followed up for 5 years. The treatment regimens in the two arms were four times community-wide MDA and two times school-based MDA alternating with 2 years without treatment. *Schistosoma mansoni*–associated morbidity was measured at baseline (year 1), year 3, and year 5, with the exception of ultrasound (US) measurements that were performed only at year 1 and year 5.

A previous article described anthropometric indices, hemoglobin (Hb) levels, physical fitness, quality of life, and US-detected organ pathology in the cohort of children aged 7–8 years at baseline.^8^ The aim of the present study was to investigate whether different morbidity markers among cohort children differ by study arm at year 5 and whether the development in morbidity markers among cohort children from baseline to follow-up differs by study arm.

## METHODS

### Study area and population.

The study was carried out from August 2011 to September 2016 in villages/schools situated within a 10-km distance from Lake Victoria in Mwanza Region of Tanzania, where transmission of *S*. *mansoni* is perennial and where a prior site selection survey had identified a prevalence of *S*. *mansoni* of ≥ 25%. At the baseline, the region had 1,139 primary schools enrolling a total of 902,367 schoolchildren, which is more than 95% of all school-aged children in the region.^9^

In two of the six arms of the SCORE intervention trial (Figure 1), four villages were randomly selected (but see restrictions in the following text) to nest a cohort of 100 first-year students (aged 7–8 years) for morbidity assessments. Thus, 800 children were included at baseline. Proper sample size calculations were not performed for the cohort study, but the largest sample possible, given the available resources, was enrolled. For the intervention trial, the two arms with a total of 50 villages were situated in Geita, Sengerema, Misungwi, Nyamagana, Ilemenau, and Magu districts. For convenience, Geita and Magu districts were excluded and the selection of the eight villages was performed in the four districts closest to Mwanza. This reduced the total number of villages to 25, from which the eight villages were randomly selected. The trial protocol is described in Shen et al.^10^ Eight villages (Bukumbi, Chamabanda, Isamilo, Kafundikile, Kasomeko, Katunguru, Nyamatongo, and Nyamazugo) were included, but unfortunately, one village (Kafundikile) had to be excluded because of low level of cooperation.

The children were examined for *S. mansoni* infection and morbidity parameters in the 1st, 3rd, and 5th years before that year’s intervention. The following morbidity indicators were measured in addition to the parasitological investigations: height, weight, Hb, physical fitness, quality of life, and US-detected organ pathology (only performed at year 1 and year 5).

### Ethics statement on subject recruitment.

The study was reviewed and approved by the Medical Research Coordination Committee of the National Institute for Medical Research (NIMR), Tanzania, (ethics clearance certificate no. NIMR/HQ/R.8a/Vol.IX/1022) and the University of Georgia Institutional Review Boards, Athens, GA (2011-10353-1). Before examination and sample collection, the reasons for the survey and the procedure of sample collection were explained to the children and the adult population in the communities, including local leaders, school administration, teachers, and health and education personnel. The trial was registered with ClinicalTrials.gov (NCT02162875) and the International Standard Randomized Controlled Trial (95819193). Only children who gave assent to participate and whose parents or guardians had given written informed consent were eligible for inclusion.

### Stool sample collection, examination, and treatment.

Participants were given stool containers and asked to return a fresh stool specimen on three consecutive days. Specimens were processed using duplicate Kato–Katz thick smears, with a 41.7-mg template^11^ from each specimen in the school, and all the slides were transported to the NIMR and examined for *S*. *mansoni* eggs. Infections with soil-transmitted helminths were not investigated as *Ascaris lumbricoides* and *Trichuris trichiura* are reported to be uncommon in this area,^12^ and the eggs of hookworms would not be visible at the time of reading. School-based treatment (SBT) was performed in the school and included both enrolled and non-enrolled school-aged children. Village health workers helped in identifying the non-enrolled children. Community-wide treatment (CWT) was offered to the whole community, with the exception of children younger than 5 years or less than 94 cm in height, and pregnant women. Treatment was performed at a central place (school, health facility, or marketplace). All treatments were performed by the study team who directly observed that tablets were taken.

### Blood collection and Hb assessments.

A fingerprick blood sample was collected from each individual, and Hb levels were measured using a portable HemoCue photometer (Ängelholm, Sweden). Hemoglobin level was reported in g/L, and final values used in the analysis were adjusted for altitude by subtracting 2 g/L from the raw values.^13^ Anemia was defined as Hb values < 115 g/L for children younger than 12 years and Hb < 120 g/L for children of 12 years and older but younger than 15 years according to the WHO guidelines.^13^

### Anthropometric measurements.

Height was measured on barefooted children using a wooden stadiometer. The child stood on the base of the stadiometer with their heels, buttocks, shoulder blades, and back of the head touching the vertical backboard and looking straight ahead. When correctly positioned, the ruler was lowered and the height was measured in centimeters with one decimal. Weight was measured on a digital scale on barefooted children having removed any excess clothing. Weight was measured in kilograms to one decimal. Height and weight were measured twice by the same examiner, and the mean was recorded. *Z*-scores were calculated using the WHO growth reference data tables for 5- to 19-year-old children.^14^ Wasting was defined as a body mass index (BMI)-for-age *Z*-score of < −2 SD, and stunting was defined as a height-for-age *Z*-score of < −2 SD.

### Physical fitness.

Physical fitness was assessed using the 20-m shuttle run fitness test (20mSRT) as described by Bustinduy et al.^15^ In brief, during the test, children run continuously between two lines 20-m apart at increasing speeds, turning when signaled to do so by recorded beeps. A “shuttle” is defined as a run from one line to the other. The running field was prepared in the school compound, runners were separated at least one meter apart, and recorders were placed at each end; every recorder was responsible for taking notes of three to five children. The recorder noted the level at which the test subject stopped and how many shuttles the child completed within that level. These numbers are correlated with a maximal oxygen uptake, the VO_2_ max in mL/kg/minutes, as described by Müller et al.^16^

### Quality of life.

Evaluation of quality of life was performed using the validated Pediatric Quality of Life Inventory 4.0 Short Form 15 (PedsQL4.0 SF15) instrument for children.^8^ The questionnaire consists of 15 questions and is divided into four parts with three to five questions each. The four parts describe four dimensions of functioning: problems with physical activities (physical), problems with feelings (emotional), problems with getting along with others (social), and problems with keeping up in school (school). The answers were scored from 0 to 4, where 0 is never, 1 is almost never, 2 is sometimes, 3 is often, and 4 is almost always. Responses were transformed to scores: 100, 75, 50, 25, and 0, respectively, resulting in a scale range from 0 to 100, with the higher score indicating a perceived better quality of life.

### Abdominal ultrasonography.

Abdominal US was performed using a portable generator-powered US machine (Aloka Sonocamera SSD-500 with a 3.5-MHz curvilinear probe, Tokyo, Japan). Examinations were performed according to the Niamey protocol^17^ by a senior sonographer with extensive experience of US examination of *S*. *mansoni*–infected individuals. Children were examined lying on their backs with their legs stretched on an examination table. Measurements involved the length of the left liver lobe (mm), spleen length (mm), portal branch thickening, and portal vein diameter (mm). The liver texture was translated into six texture patterns, A–F, as described in the Niamey protocol. Image patterns A and B were considered normal. Image patterns C and D were considered mild and moderate periportal fibrosis (PPF), respectively, whereas liver patterns E and F were considered advanced PPF. Hepatomegaly (enlarged liver), splenomegaly (enlarged spleen), and increased PVD were defined as two SDs above standard reference measurements for healthy uninfected children in corresponding height groups.^17,18^

### Statistical analysis.

Data were analyzed using IBM statistics SPSS version 24 (IBM, Armonk, NY). A person was considered positive for infection if at least one egg was found in any of the six slides. The mean egg count of the six slides was calculated and multiplied by 24 to express the intensity as eggs per gram (epg) of stool. In case slides were missing, the calculation was performed on available slides. Group intensity was reported as the arithmetic mean of epg from the total number of investigated persons (village-level intensity) or of the infected persons only (individual-level intensity).

As village is the clustering factor, all mean prevalence was calculated as the means of the prevalence of individual villages. Likewise, all means and scores were calculated as the means of the mean/scores of individual villages. As all analyses had two independent variables, time/year as the within-subject variable and treatment regimen/arm as the between-subject variable, the two-way mixed-model ANOVA was used to analyze whether there was an effect of year and/or arm on parasitological parameters and morbidity markers. *P*-values of less than 0.05 were considered statistically significant.

## RESULTS

Because one village (Kafundikile) was excluded from the study (mentioned earlier), analyses were performed on data from seven villages from where 572 children aged 7–8 years participated in the baseline survey. In year 3, it was possible to retrieve 459 of the cohort children, and in year 5, 374 of the children were found. Thus, 198 children were lost to follow-up during the 5-year period. Table 1 shows the year 1 (baseline), year 3, and year 5 characteristics of participants for the two arms and the characteristics of children lost to follow-up. At baseline, there were no significant differences in any of the characteristics recorded between those children who were lost to follow-up and those who were followed up in year 3 and year 5.

**Table 1:** Baseline (year 1), year 3, and year 5 characteristics of participants, and loss to follow-up, by arm

Treatment arm*	Year 1 (baseline), *N* = 572		Year 3, *N* = 459		Year 5, *N* = 374		Baseline characteristics of children lost to follow-up at year 5, *N* = 198	
	CWT arm	SBT arm	CWT arm	SBT arm	CWT arm	SBT arm	CWT arm	SBT arm
Number of villages	3	4	3	4	3	4	3	4
Number in cohort	254	318	196	263	153	221	101	97
% Female (*n*)	57.9 (147)	51.3 (163)	58.7 (115)	51.7 (136)	60.1 (92)	52.5 (116)	54.5 (55)	48.5 (47)
Mean age (SD)	7.3 (0.5)	7.5 (0.5)	9.6 (0.5)	9.7 (0.5)	11.3 (0.5)	11.6 (0.5)	7.3 (0.5)	7.6 (0.5)
Number tested for schistosomiasis	254	318	196	263	153	221	101	97
Number infected	175	161	62	87	56	97	68	42
Mean prevalence, % (SD)†	66.6 (31.8)	52.0 (28.0)	29.5 (17.1)	33.6 (20.0)	35.3 (18.8)	46.5 (39.1)	67.9 (29.5)	47.0 (28.0)
Village-level arithmetic mean infection intensity, epg (SD)‡	130.7 (99.3)	116.9 (185.0)	13.8 (12.9)	16.1 (21.1)	17.5 (15.7)	25.2 (29.0)	118.3 (94.6)	158.3 (265.6)
Individual-level arithmetic mean infection intensity, epg (SD)§	167.8 (99.1)	152.8 (180.8)	41.5 (25.9)	36.3 (26.5)	44.9 (22.9)	36.3 (30.3)	155.2 (116.4)	214.0 (275.4)

* CWT arm = four times community-wide treatment; SBT arm = twice school-based treatment alternating with years without treatment.

† Means of the prevalence of individual village.

‡ Mean of the means of individual villages, all investigated children included.

§ Mean of the means of individual villages, only infected children included.

Table 2 and Supplemental Figure 1 present the mean prevalence, mean village-level intensity, and mean individual-level intensity for those 374 children who were present at years 1, 3, and 5. Table 2 also presents the absolute and relative difference of prevalence and intensities between year 5 and year 1. In a two-way mixed-model ANOVA, there was a significant difference in individual-level infection intensity from year 1 to year 5 (*P* = 0.041), but there was no effect of study arm. Prevalence and village-level intensity did not differ by arm or by year.

**Table 2:** Year 1/year 5 comparison of prevalence and intensity, by arm, in a cohort of 374 children (153 in CWT arm and 221 in SBT arm)

Treatment arm§	Prevalence*		Village-level intensity†		Individual-level intensity‡	
	CWT arm	SBT arm	CWT arm	SBT arm	CWT arm	SBT arm
Year 1; baseline (SD)	65.4% (34.0)	54.5% (28.1)	139.0 epg (113.0)	103.6 epg (158.5)	177.9 epg (100.2)	133.2 epg (152.3)
Year 3 (SD)	31.2% (20.4)	33.1% (20.2)	16.1 epg (17.1)	16.5 epg (22.8)	39.5 epg (36.8)	36.1 epg (30.7)
Year 5 (SD)	35.3% (18.8)	46.5% (39.1)	17.5 epg (15.7)	25.2 epg (29.0)	44.9 epg (22.9)	36.3 epg (30.3)
Absolute difference between year 5 and baseline	30.1%	8.0%	121.5 epg	78.4 epg	133.0 epg	96.9 epg
Relative difference between year 5 and baseline (% change)	46.0	14.7	87.4	75.7	74.8	72.7
ANOVA table‖						
Effect of arm (CWT vs. SBT)	*F*_(df1)_ = 0.000, *P* = 0.99		*F*_(df1)_ = 0.049, *P* = 0.83		*F*_(df1)_ = 0.193, *P* = 0.68	
Effect of year (year 1 vs. year 5)	*F*_(df1)_ = 4.436, *P* = 0.089		*F*_(df1)_ = 4.607, *P* = 0.085		***F***_**(df1)**_ **= 7.467, *P* = 0.041**	
Effect of interaction (arm × year)	*F*_(df1)_ = 1.496, *P* = 0.28		*F*_(df1)_ = 0.215, *P* = 0.66		*F*_(df1)_ = 0.184, *P* = 0.69	

Bold value indicates statistical significant difference.

* Means of the prevalence of individual village.

† Mean of the means of individual villages, all investigated children included.

‡ Mean of the means of individual villages, only infected children included.

§ CWT arm = four times community-wide treatment; SBT arm = twice school-based treatment alternating with years without treatment.

‖ Two-way mixed-model ANOVA.

Table 3 presents the results on morbidity markers, by arm and by year, whereas Table 4 presents the statistical analysis of the data presented in Table 3. The tables show a significant decrease in anemia and a significant increase in Hb from year 1 to year 5, irrespective of the study arm. There was a significant decrease in VO_2_ max from year 1 to year 5. The prevalence of hepatomegaly was significantly reduced from year 1 to year 5 and was significantly more in the CWT arm than in the SBT arm. The findings presented in Table 3 are illustrated in Supplemental Figures S1–S4.

**Table 3:** Morbidity markers by arm and by year in a cohort of 374 children

	CWT arm (*n* = 153, 3 villages)* percent/value			SBT arm (*n* = 221, 4 villages)* percent/value		
	Year 1	Year 3	Year 5	Year 1	Year 3	Year 5
Prevalence of stunting, % (SD)†	12.4 (4.9)	13.6 (9.8)	15.7 (0.4)	7.7 (5.2)	11.7 (11.1)	22.6 (17.7)
Prevalence of wasting, % (SD)‡	11.2 (15.2)	3.6 (4.7)	25.5 (29.0)	7.7 (2.2)	1.5 (2.0)	2.7 (2.3)
Mean Hb, gm/L (SD)	117.6 (3.8)	116.3 (1.4)	122.4 (0.8)	118.5 (2.6)	115.5 (6.3)	125.9 (5.6)
Prevalence of anemia, % (SD)	40.2 (9.1)	39.2 (5.2)	26.2 (6.4)	36.6 (6.1)	40.4 (13.9)	22.7 (12.5)
Mean VO_2_ max score (SD)	51.7 (1.5)	46.3 (1.8)	48.5 (3.5)	51.2 (1.5)	46.6 (1.5)	47.9 (1.7)
Mean PedsQL score (SD)						
Total score	75.8 (22.3)	80.3 (2.7)	82.1 (9.1)	83.2 (17.5)	82.4 (1.3)	84.8 (5.7)
Physical	78.8 (29.0)	90.4 (4.5)	88.3 (12.0)	84.2 (23.0)	92.0 (3.2)	89.8 (6.7)
Emotional	71.9 (19.5)	71.9 (3.1)	76.2 (7.2)	81.0 (12.7)	77.3 (1.7)	76.7 (9.7)
Social	77.9 (24.7)	76.9 (2.1)	80.5 (7.9)	85.2 (19.4)	74.9 (0.6)	87.5 (4.9)
School	71.9 (20.8)	78.7 (1.3)	81.4 (10.3)	82.8 (14.6)	80.8 (2.7)	84.6 (5.2)
Hepatomegaly, % (SD)§	70.2 (1.6)	–	51.0 (6.0)	77.5 (3.0)	–	63.3 (7.0)
Splenomegaly, % (SD)	17.4 (15.0)	–	17.0 (7.7)	31.3 (19.0)	–	32.1 (15.0)
Hepatosplenomegaly, % (SD)	16.0 (13.6)	–	12.6 (6.4)	25.7 (16.2)	–	24.6 (9.2)
Enlarged portal vein, % (SD)	6.2 (4.2)	–	14.8 (2.3)	7.4 (7.6)	–	14.9 (10.2)
Liver pattern						
B, *n* (%)	1 (0.7)	–	2 (1.3)	1 (0.5)	–	5 (2.3)
C or higher, *n* (%)	1 (0.7)	–	1 (0.7)	3 (1.4)	–	2 (0.9)

* CWT arm = four times community-wide treatment; SBT arm = twice school-based treatment alternating with years without treatment. Percent and values represent the mean of the prevalence and the mean of the means of individual villages, respectively.

† Stunting = height-for-age *Z* < −2SD.

‡ Wasting = BMI-for-age *Z* < −2SD.

§ Ultrasound investigations were not performed at year 3.

**Table 4:** Year 1/year 5 comparison of morbidity markers using the two-way mixed-model ANOVA in a cohort of 374 children

	Stunting (%)*	Wasting (%)†	Hb (gm/L)	Anemia (%)	VO_2_ max (mL/kg/minutes)
Effect of arm (CWT vs. SBT)	*F*_(df1)_ = 0.021, *P* = 0.89	*F*_(df1)_ = 5.973, *P* = 0.058	*F*_(df1)_ = 0.823, *P* = 0.41	*F*_(df1)_ = 0.566, *P* = 0.49	*F*_(df1)_ = 0.230, *P* = 0.65
Effect of year (year 1 vs. year 5)	*F*_(df1)_ = 2.83, *P* = 0.15	*F*_(df1)_ = 0.217, *P* = 0.66	***F***_**(df1)**_ **= 12.958, *P* = 0.016**	***F***_**(df1)**_ **= 7.580, *P* = 0.040**	***F***_**(df1)**_ **= 8.997, *P* = 0.030**
Effect of interaction (arm × year)	*F*_(df1)_ = 1.208, *P* = 0.32	*F*_(df1)_ = 0.944, *P* = 0.38	*F*_(df1)_ = 0.569, *P* = 0.49	*F*_(df1)_ = 0.000, *P* = 0.99	*F*_(df1)_ = 0.005, *P* = 0.95

	Physical PedsQL (score)	Emotional PedsQL (score)	Social PedsQL (score)	School PedsQL (score)	Total PedsQL (score)
Effect of arm (CWT vs. SBT)	*F*_(df1)_ = 0.201, *P* = 0.67	*F*_(df1)_ = 0.482, *P* = 0.52	*F*_(df1)_ = 1.206, *P* = 0.32	*F*_(df1)_ = 1.257, *P* = 0.31	*F*_(df1)_ = 0.612, *P* = 0.47
Effect of year (year 1 vs. year 5)	*F*_(df1)_ = 0.360, *P* = 0.57	*F*_(df1)_ = 0.000, *P* = 0.99	*F*_(df1)_ = 0.058, *P* = 0.82	*F*_(df1)_ = 0.485, *P* = 0.52	*F*_(df1)_ = 0.181, *P* = 0.69
Effect of interaction (arm × year)	*F*_(df1)_ = 0.025, *P* = 0.88	*F*_(df1)_ = 0.393, *P* = 0.56	*F*_(df1)_ = 0.000, *P* = 0.99	*F*_(df1)_ = 0.228, *P* = 0.65	*F*_(df1)_ = 0.067, *P* = 0.81

	Hepatomegaly (%)	Splenomegaly (%)	Hepatosplenomegaly (%)	Enlarged PVD (%)
Effect of arm (CWT vs. SBT)	***F***_**(df1)**_ **= 9.333, *P* = 0.028**	*F*_(df1)_ = 2.500, *P* = 0.18	*F*_(df1)_ = 2.217, *P* = 0.20	*F*_(df1)_ = 0.027, *P* = 0.88	
Effect of year (year 1 vs. year 5)	***F***_**(df1)**_ **= 65.157, *P* < 0.0005**	*F*_(df1)_ = 0.001, *P* = 0.98	*F*_(df1)_ = 0.145, *P* = 0.72	*F*_(df1)_ = 4.030, *P* = 0.10	
Effect of interaction (arm × year)	*F*_(df1)_ = 1.412, *P* = 0.29	*F*_(df1)_ = 0.007, *P* = 0.94	*F*_(df1)_ = 0.038, *P* = 0.85	*F*_(df1)_ = 0.017, *P* = 0.90	

Bold values indicate statistical significant differences.

* Stunting = height-for-age *Z* < −2SD.

† Wasting = BMI-for-age *Z* < −2SD.

## DISCUSSION

In this cohort study, we compared the impact of two different MDA strategies over time on *S. mansoni* infection and related morbidity parameters in schoolchildren. Both treatment regimens decreased the prevalence and intensity of *S. mansoni* infection from baseline to year 5, although this was only significant for the mean individual-level intensity. There was no difference between the two treatment approaches in the impact on infection prevalence and intensity. Both treatment approaches significantly increased Hb levels and decreased the proportion of anemic children, but with no difference between the study arms. Physical fitness also decreased significantly from baseline to year 5 in both arms, with no difference between the arms. In contrast to this, the proportion of children with hepatomegaly decreased in both treatment arms and significantly more in the CWT approach than in the SBT approach. For all other characteristics, there were no significant changes by year or by study arm.

Physical fitness decreased significantly from baseline to year 5, which is consistent with the findings from a study in Kenya, where a decrease in VO_2_ max scores was seen in children from the age of 5 to 18 years.^15^ The Kenyan authors related this to an increase in anemia, stunting, and wasting due to chronic parasitic infections. The same development in VO_2_ max was reported in a parallel SCORE study in Kenya, where the authors suggest that this could be due to an increase in the prevalence of malaria and anemia in the first 2 years of the study.^19^ However, in the present study, there was an improvement in Hb and reduction in anemia, and, therefore, we suggest that the decrease could be caused by an increase in the body weight of the children. Although the absolute maximal oxygen consumption normally rises with age, the VO_2_ max (maximum oxygen uptake per kilo of body weight, mL/kg/minutes) actually can decrease as children increase their body mass as a result of growth. This is evident, particularly in girls, also in countries without chronic parasitic infections such as Canada.^15^

Unfortunately, we only investigated the children for malaria in year 3, so we have no data from the cohort in years 1 and 5. However, the overall trend in the area is that malaria has decreased because of scaling up of the use of insecticide-treated nets and indoor residual spraying. If the cohort children experienced such a reduction in malaria from year 1 to year 5, this could have contributed to the improvement of Hb and reduction in the number of children with anemia.

The present study was mirrored by another SCORE study in Kenya also along Lake Victoria and with a prevalence of ≥ 25% *S. mansoni* among schoolchildren.^19^ The Kenyan study used the same methodology and the same MDA approaches as the present study in Tanzania. In contrast with the present study, Kenyan schoolchildren were characterized by higher prevalence and intensity of infection, more anemia, and stunting as well as more pronounced hepatosplenic morbidity at the baseline.^20^ At year 5, the Kenyan cohort children showed an almost unchanged prevalence of infection and high-intensity infections in both the study arms. However, there was a significant reduction in wasting, hepatosplenic pathology, and improved quality of life scores. Children without infection were less stunted than those still infected at year 5. None of the changes were associated with the study arm.

The differences in the two cohort populations regarding the effect of interventions were examined during a comparative study focusing on socioeconomic and dietary differences between the two study populations.^21^ The comparative study showed that there were significant differences in dietary intake between schoolchildren in Kenya and Tanzania, with Tanzanian children eating a much larger amount of protein-rich diet (fish) on a daily basis than their Kenyan counterparts. The dietary differences between the two study populations might well explain the higher prevalence of wasting and stunting among Kenyan schoolchildren. This could also explain the higher prevalence of anemia and lower levels of physical fitness in Kenyan children.^21^

Although both countries were high-transmission countries with a prevalence of *S. mansoni* ≥ 25%, the prevalence of infection was higher in Kenyan study participants than in Tanzanian participants and, especially, the intensity of infection was higher in Kenya, with more children having high-intensity infections. Previous studies in Uganda have shown that liver and spleen pathology is only seen in children with very high infection intensities.^22,23^ In the Tanzanian study participants, the combination of good nutritional status and low intensity of *S. mansoni* infection may explain the lower level of schistosomiasis-related morbidity and the modest impact of the two MDA strategies. Furthermore, the cluster randomized design making villages the unit of measurement reduced the power to detect differences in infection and morbidity parameters in case real differences existed.

In conclusion, the results show that in this study population, the impact of an intensive community-based MDA was significantly better than that of a school-based strategy only with regard to reduction in hepatomegaly. Other parameters that improved, such as individual-level infection intensity, Hb level, and anemia, were associated with years of intervention and not the MDA approach.

## Supplemental figures

Supplemental materials
