# Supplementary material for: Changes in Morbidity, Physical Fitness, and Perceived Quality of Life among Schoolchildren following Four Years of Different Mass Drug Administration Strategies against Schistosoma mansoni Infection in Mwanza Region, Northwestern Tanzania
Source: Am J Trop Med Hyg. 2019 Nov 11;102(1):100–5. doi: 10.4269/ajtmh.19-0428 (PMC6947808; doi:10.4269/ajtmh.19-0428)
Supplement: Supplementary file 1 [file tpmd190428.SD1.pdf]

The following are supplemental materials and will be published online only

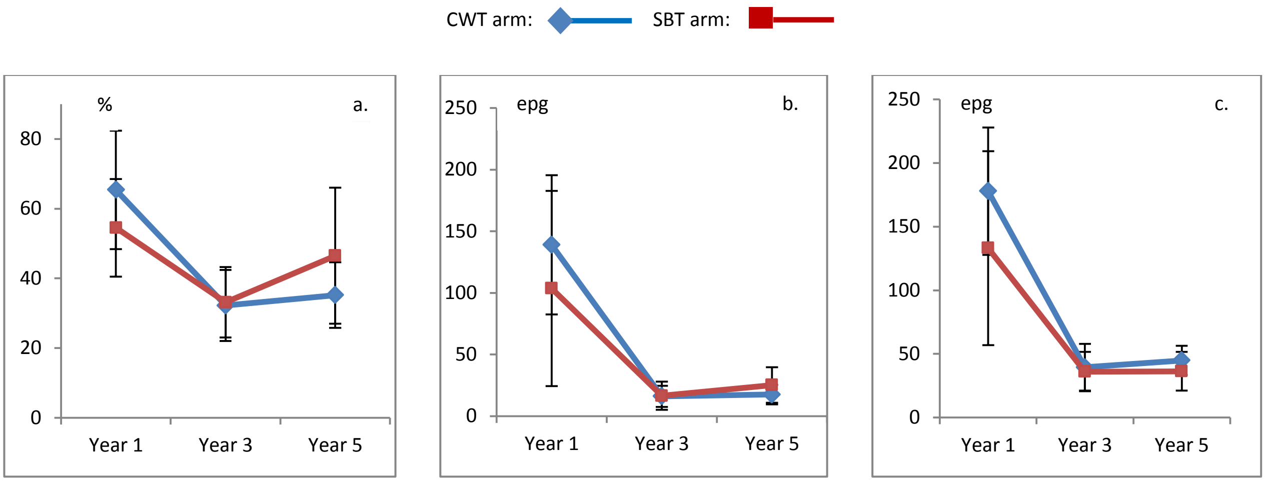

Supplemental Figure S1. Prevalence of *Schistosoma mansoni* infection (a.), village-level mean intensity (b.), and individual-level mean intensity (c.) in year 1, 3 and 5 in the community-wide treatment (CWT) and school-based treatment (SBT) arms; error bars indicate standard deviation; epg = eggs per gram feces

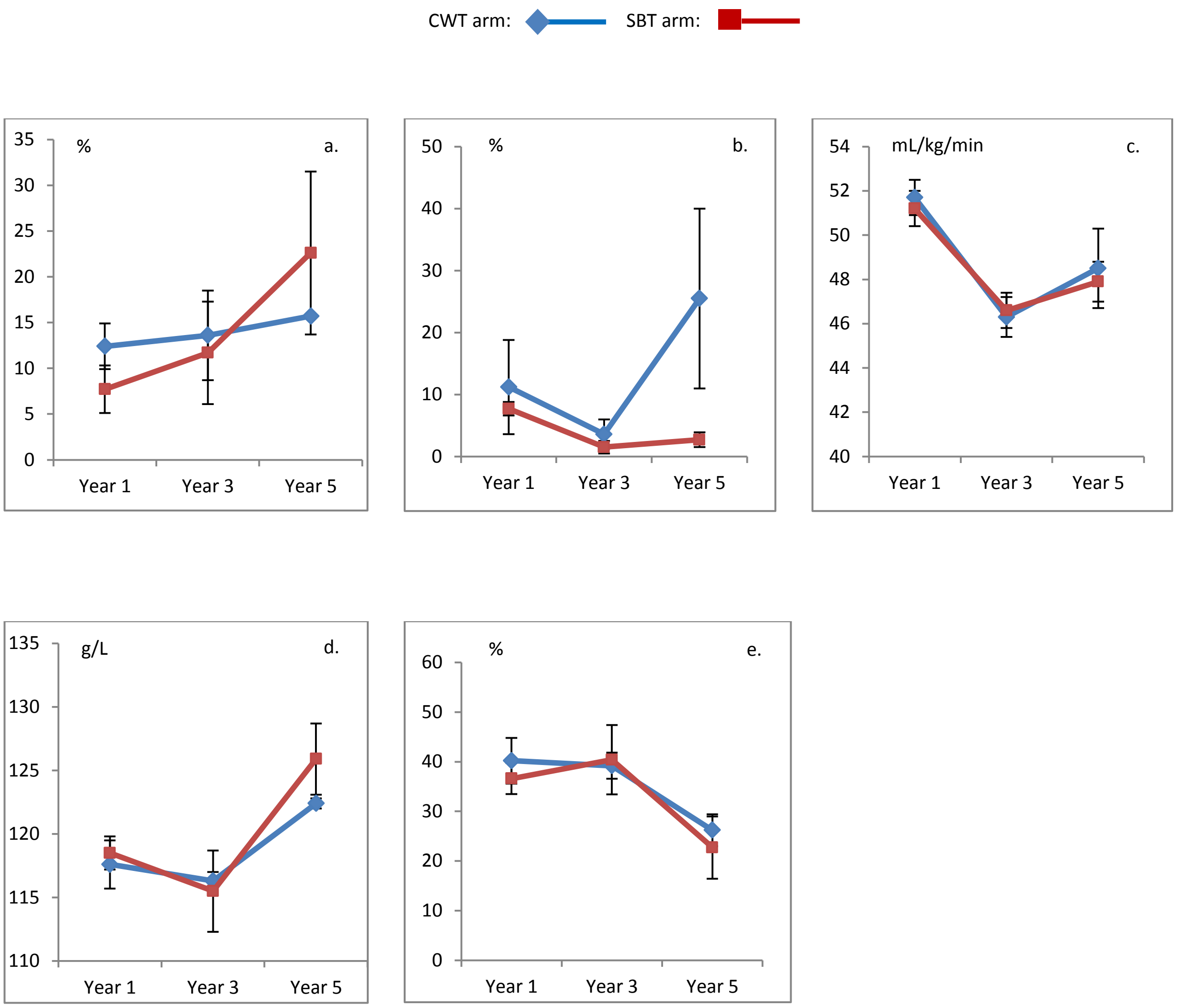

Supplemental Figure S2. Prevalence of stunting (height-for-age Z-score of < −2 SD) (a.) and wasting (BMI-for-age Z-score of < −2 SD) (b.), physical fitness as maximal oxygen uptake (VO_2 max) (c.), hemoglobin level (d.), and prevalence of anemia (e.) in year 1, 3 and 5 in the community-wide treatment (CWT) and school-based treatment (SBT) arms; error bars indicate standard deviation

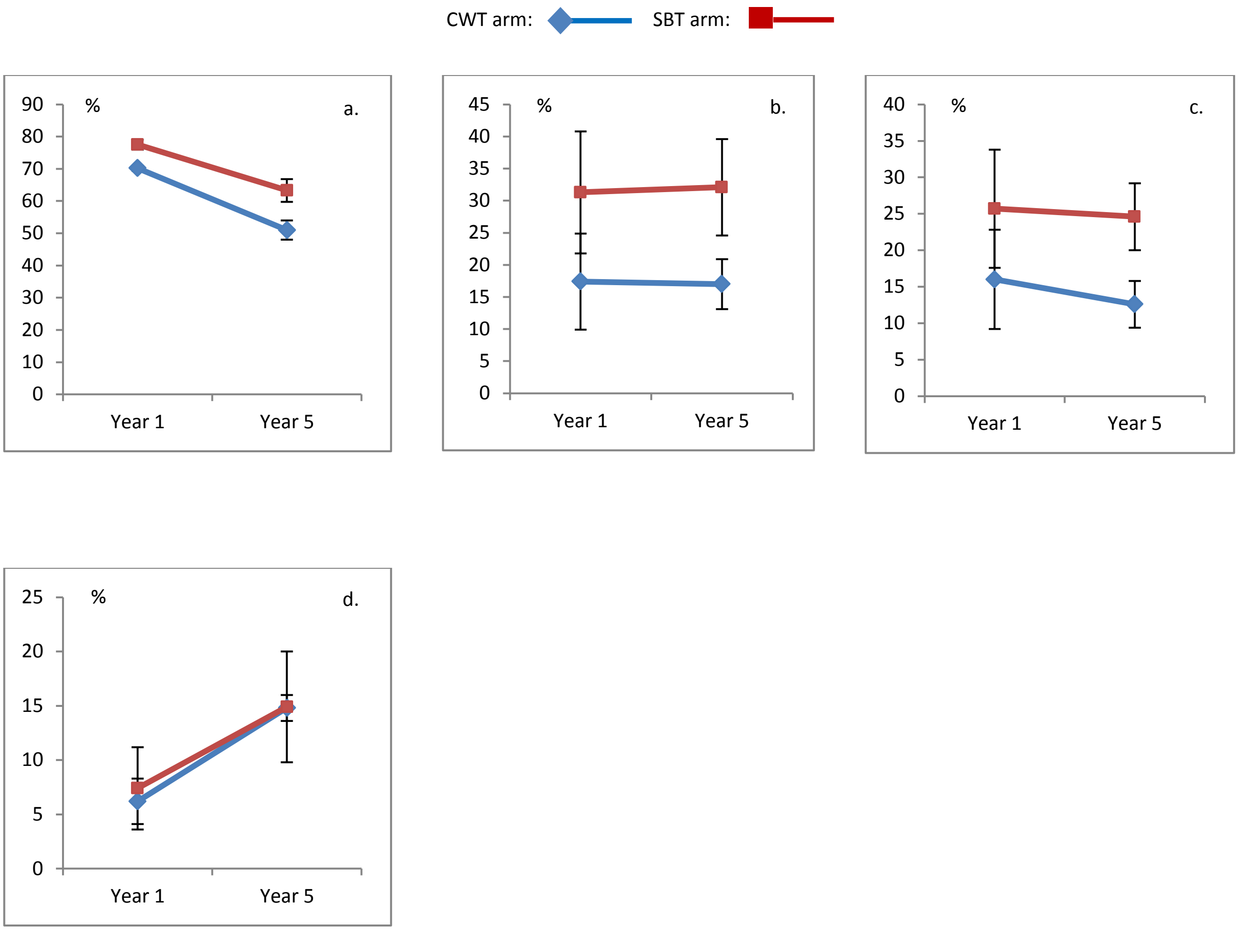

Supplemental Figure S3. Prevalence of hepatomegaly (a.), splenomegaly (b.), hepatosplenomegaly (c.), and enlarged portal vein diameter (d.) in year 1 and 5 in the community-wide treatment (CWT) and school-based treatment (SBT) arms; error bars indicate standard deviation

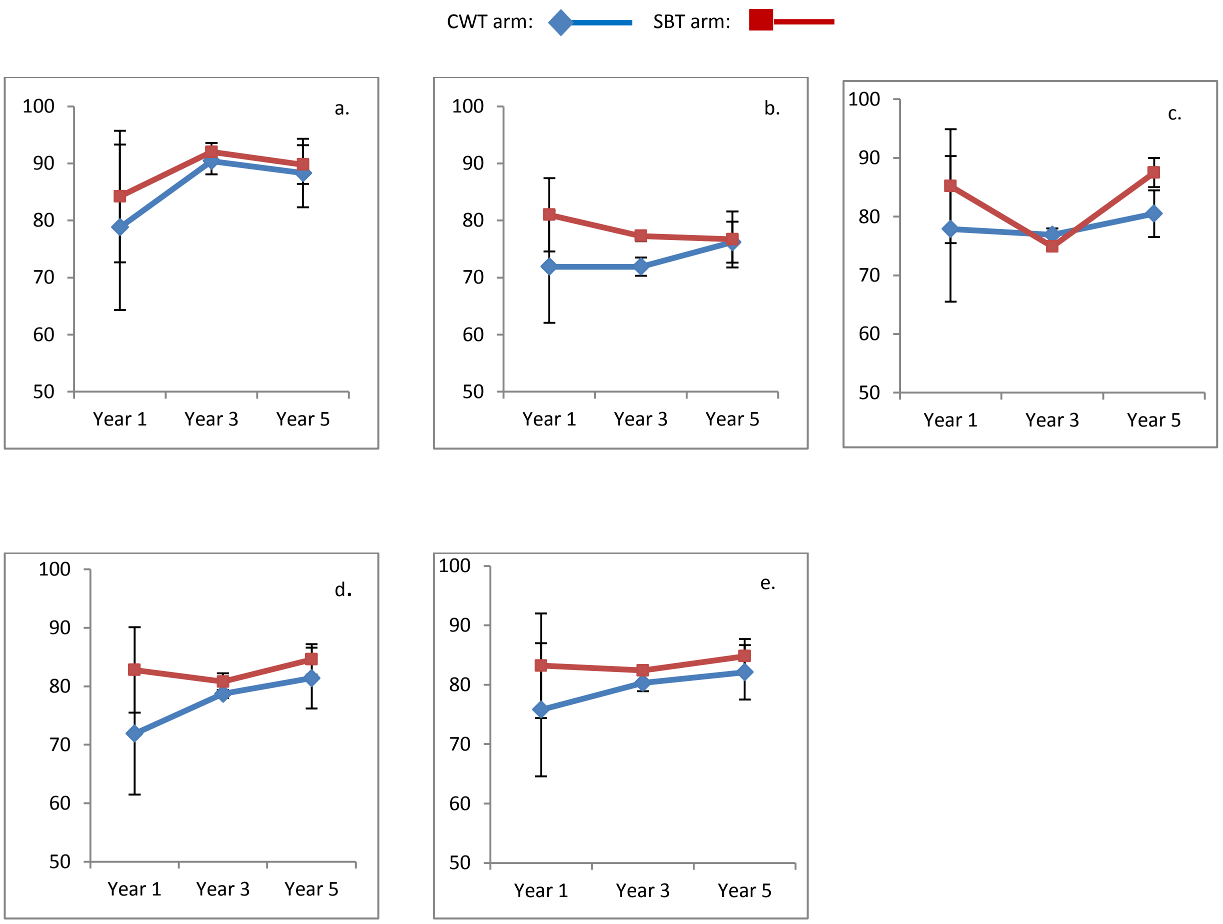

Supplemental Figure S4. Pediatric quality-of-life (PedsQL) scores from 0 to 100 in year 1, 3 and 5 in the community-wide treatment (CWT) and school-based treatment (SBT) arms in the four categories of physical (a.), emotional (b.), social (c.), school (d.), and total score (e.); error bars indicate standard deviation
